# Visualization of species pairwise associations: a case study of surrogacy in bird assemblages

**DOI:** 10.1002/ece3.1182

**Published:** 2014-07-29

**Authors:** Peter W Lane, David B Lindenmayer, Philip S Barton, Wade Blanchard, Martin J Westgate

**Affiliations:** Fenner School of Environment and Society, ARC Centre of Excellence for Environmental Decisions, National Environmental Research Program, The Australian National UniversityCanberra, Australian Capital Territory, 0200, Australia

**Keywords:** Community, competition, interactions, likelihood ratio, mutualism, odds ratio

## Abstract

Quantifying and visualizing species associations are important to many areas of ecology and conservation biology. Species networks are one way to analyze species associations, with a growing number of applications such as food webs, nesting webs, plant–animal mutualisms, and interlinked extinctions. We present a new method for assessing and visualizing patterns of co-occurrence of species. The method depicts interactions and associations in an analogous way with existing network diagrams for studying pollination and trophic interactions, but adds the assessment of sign, strength, and direction of the associations. This provides a distinct advantage over existing methods of quantifying and visualizing co-occurrence. We demonstrate the utility of our new approach by showing differences in associations among woodland bird species found in different habitats and by illustrating the way these can be interpreted in terms of underlying ecological mechanisms. Our new method is computationally feasible for large assemblages and provides readily interpretable effects with standard errors. It has wide applications for quantifying species associations within ecological communities, examining questions about particular species that occur with others, and how their associations can determine the structure and composition of communities.

## Introduction

Understanding why species occur where they do has been a fundamental part of ecology since the inception of the discipline (Elton 1927). A key part of species distribution studies has been to quantify the composition of assemblages of taxa (e.g., Putman 1994; Magurran and McGill 2011). The identity, abundance, and co-occurrence of multiple species are what defines and distinguishes ecological communities, and therefore, methods to examine and visualize sets of co-occurring and interacting species are important in the studies of ecosystems.

Many approaches have been developed to show when particular sets of species occur in some places but not others (e.g., Digby and Kempton 1987; McCune et al. 2002; Duchamp and Swihart 2008), and they have indicated several influential factors. These include biogeographic history, overlapping ranges, shared responses to habitat suitability, and the influence of one species on another (Godsoe and Harmon 2012) such as through predator–prey relationships (Krebs et al. 2001; Estes et al. 2011), mutualisms (Bascompte and Jordano 2007; Bascompte 2009), and competition (Mac Nally et al. 2012).

Understanding the associations, and thus potential interactions, between species in an assemblage is important to many rapidly expanding fields, including food webs (Tylianakis 2008; Saterberg et al. 2013), nesting webs (Martin et al. 2004), ecological networks of plant–animal mutualisms (Bascompte et al. 2003; Bastolla et al. 2009), and interlinked extinctions (Saterberg et al. 2013). Moreover, several studies recognize the need to conserve not only species themselves, but also the associations between species as these are also critical in the functioning and assembly of ecological communities (e.g., Tylianakis et al. 2010). Key to the advancement of these fields is the assessment of the sign of associations between species (positive or negative effect of one species on the presence of another) and quantification of the strength of those associations. Few studies have attempted to examine these aspects of species co-occurrence (but, e.g., see Ovaskainen et al. 2010; Steele et al. 2011), thus limiting our understanding of species interaction and association networks.

In this study, we present a new method for examining and visualizing multiple pairwise associations within diverse assemblages. Our approach goes beyond examining the identity of species or the presence of associations in an assemblage by identifying the sign and quantifying the strength of associations between species. In addition, it establishes the direction of associations, in the sense of which individual species tends to predict the presence of another. This additional information enables assessments of mechanisms giving rise to observed patterns of co-occurrence, which several authors have suggested is a key knowledge gap (reviewed by Bascompte 2010).

We demonstrate the value of our approach using a case study of bird assemblages in Australian temperate woodlands. This is one of the most heavily modified ecosystems worldwide, where understanding changes in assemblage composition is of significant interest (Lindenmayer et al. 2010). We use an extensive longitudinal dataset gathered from more than a decade of repeated surveys of birds on 199 patches of remnant native woodland (remnants) and of revegetated woodland (plantings). To demonstrate the value of our approach, we first assess the co-occurrence patterns of species in remnants and then contrast these with the patterns in plantings.

Our new method has wide applications for quantifying species associations within an assemblage, examining questions related to why particular species occur with others, and how their associations can determine the structure and composition of whole assemblages.

### Measurement and visualization of species pairwise associations

Our approach for examining species pairwise association seeks to quantify the strength of association between two individual species in terms of two odds ratios: the odds of the first species being present when the second one is (i.e., *P*/(1−*P*), where *P* is the probability of the first species being present when the second one is), divided by the odds of the first species occurring regardless of the second; and vice versa. The first odds ratio is a measure of how effective the second species is as an indicator of the presence of the first (or as an indicator of absence, if the odds ratio is <1). An odds ratio is more appropriate than either a probability ratio or difference because it takes account of the limited range of percentages (0–100%): any given value of an odds ratio approximates to a multiplicative effect on rare percentages of presence, and equally on rare percentages of absence, and cannot give invalid percentages when applied to any baseline value. Moreover, such an application to a baseline percentage is straightforward, giving a readily interpretable effect in terms of change in percentage presence. This pair of odds ratios is also more appropriate for our purposes than a single odds ratio, calculated as above for either species as first but with the denominator being the odds of the first species occurring when the second does not. That ratio is symmetric (it gives the same result whichever species is taken first) and does not take account of how common or rare each species is (see below) and hence the potential usefulness of one species as a predictor of the other. For the illustrative example in Table 1, our odds ratio for indication of Species A by Species B is (15/5)/(50/50) = 3 and of B by A is (15/35)/(20/80) = 1.71. These correspond to an increase in presence from 50 to 75% for Species A, if Species B is known to occur, but only an increase from 20 to 30% for Species B if Species A is known to occur. The symmetric odds ratio is (15/5)/(35/45) = (15/35)/(5/45) = 3.86, which gives the same importance to both of these increases.

**Table 1:** Schematic and illustrative two-way tables of the number of surveys in which each of two species was present or absent. Letters *c, d, e, and f* represent percentages of sites at which the two species were present or absent

	Species B		
	
Species A	Present	Absent	Total
Present	*c*	*d*	*c + d*
Absent	*e*	*f*	*e + f*
Total	*c + e*	*d + f*	*c + d + e + f*

	Species B		
	
Species A	Present	Absent	Total
Present	15	35	50
Absent	5	45	50
Total	20	80	100

For the purposes of this study, we interpret an odds ratio greater than 3 or less than ⅓ as indicating an ecologically “substantial” association. This is inevitably an arbitrary criterion, and other values can of course be used, but we consider that it corresponds to strong positive or negative associations. In terms of percentages, an odds ratio of 3 corresponds to any of the following changes: from 10 to 25%, 25 to 50%, 50 to 75%, or 75 to 90%. Conversely, an odds ratio of ⅓ corresponds to any of those changes reversed (e.g., 25 to 10%).

We use the term “indicated,” as in “Species A indicated Species B,” to mean that the odds ratio for the presence of Species B, with respect to the presence of Species A, was >3. Conversely, we use “contraindicated” to mean that the odds ratio was <⅓. In using such terms, we do not imply causality, which cannot be inferred from observational studies like ours. Note that the two odds ratios for each association are equal if (and only if) the two species are equally common across the sites or do not co-occur at all. One property of the measure is that if one species is common (>50% presence), it is not possible for it to indicate a species with less than half the presence rate of the common species, although the reverse is possible. Two species can contraindicate each other however common one of them is (unless one is ubiquitous) and certainly will do so if they do not co-occur at all. It is not possible for A to indicate B, and B to contraindicate A.

In our case study, we concentrated on those species that were “not rare” across our range of sites (observed in at least 10% of surveys). In addition, in analyses of subsets of surveys, we assessed the association between two species only if both occurred in 10% of those surveys.

**Figure 1:**
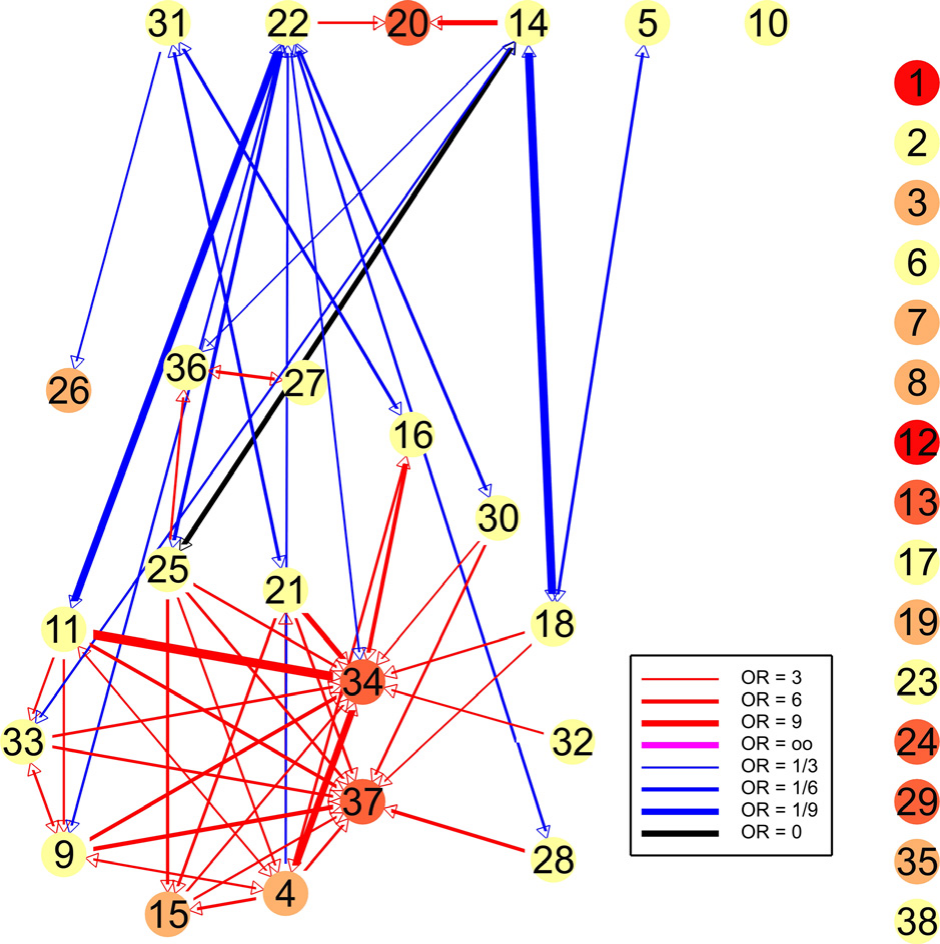
Association diagram for remnant sites (795 surveys); colored circles represent species (reference numbers identified in Table 2): red >75% presence, orange 50–75%, light brown 25–50%, yellow 10–25%, green 3.6–10%, blue < 3.6%; red arrows represent indication (thickness proportional to odds ratio) of one species by another (colored magenta if odds ratio is infinite); blue arrows similarly represent contraindication (colored black if odds ratio is 0).

We constructed an association diagram to display the pattern of association between species (e.g., Fig. 1). The nodes represent species and are color-coded according to overall presence; the edges (the lines in the diagram) represent indications (red) and contraindications (blue), with arrows indicating direction, and line thickness representing the strength of the association (the larger of the two, if there are indications or contraindications in both directions). The spatial arrangement of points (representing species) in our association diagram is derived from the strategy detailed in Appendix 1. We drew our figures using GenStat, with manual arrangement of the points to illustrate our discussion, but have also developed an R function which arranges points automatically (see R package and worked example at https://github.com/mjwestgate/sppairs).

## Comparison with existing methods

Network diagrams are used in many applications to display relationships between a set of units (Proulx et al. 2005; Mersch et al. 2013) and are employed in ecology particularly to display interactions between plants and their pollinators (Bascompte and Jordano 2007), and predators and prey (Dexter et al. 2013). However, we have seen few examples where the network represents co-occurrence within a taxonomic group (although see Pollock et al. 2014) and none where the links in the network represent odds ratios.

### Similarity coefficients

Steele et al. (2011) constructed networks with nodes representing the abundance of marine bacteria, archaea, and protists, and measurements of the marine environment. The edges represent correlations, distinguishing between positive and negative, and also between lagged and unlagged correlations over time. The correlations are formed from normalized ranked data and are referred to as local similarity coefficients, so are not readily interpretable in terms of changes in species presence; moreover, there is no concept of direction of an association, because correlations are symmetric.

### Multivariate logistic regression

Ovaskainen et al. (2010) used multivariate logistic regression to investigate interactions between fungal species, quantifying them in terms of correlations on the logistic scale. The estimates were displayed in a grid, with the size of a symbol in each cell representing the size of the correlation, using color to distinguish positive from negative correlation. Again, the correlations provide little information on species presence, and there is no concept of direction of association. Ovaskainen et al. (2010) use a Bayesian method developed by O'Brien and Dunson (2004) to fit their model for 14 species, which involves a Markov chain Monte Carlo (MCMC) algorithm. We tried this method on our data with 38 species (without the extra complication of estimating a random effect of sites) and found that 90 h of computing time was required on a laptop to run 100,000 iterations of the MCMC sampler. We also tried estimating each correlation separately (as we did to estimate our odds ratios using GLMM), which gave reliable estimates, but each pair of species required roughly 33 min to complete 10,000 iterations of the MCMC sampler (our method required just over 6 min to estimate all 1406 odds ratios). There was therefore no possibility of studying the null behavior of these estimates with simulation, as we did with our estimates of species pairwise associations. We also tried the BayesComm package (Golding 2014), which fits a similar model to that of O'Brien and Dunson (2004), but using a probit rather a logit link. This was much faster, as it is partially written in C rather than all in the R language. Pollock et al. (2014) produce network diagrams based on the O'Brien and Dunson (2004) method, again using correlation rather than asymmetric odds ratios.

### Ordination

Ordination is commonly used to study sets of ecological units (such as species) and to provide a visualization of the relationship between the units (a biplot). This could be done in our example using a metric like Bray–Curtis dissimilarity to express the relatedness of species across the sites. The resulting ordination would differ from our method for exploring species pairwise associations in several crucial respects: it would provide a single “distance” between each pair of species, rather than a pair of measures; distances do not easily discriminate between indication and contraindication; it does not provide estimates of the variability of the distance measure; and visualization unnecessarily forces the points into a Euclidean representation.

### Sensitivity and specificity

The odds ratio of the presence of Species B in relation to the presence of Species A can be seen as a property of the two-way table of presence shown in Table 1:

OR = Odds (Species B | Species A) / Odds (Species B) = {c/d} / {(*c* + *e*) / (*d* + *f*)}

This can be re-expressed as {*c* / (*c* + *e*)} / {*d*/(*d* + *f*)}, which is the ratio of the sensitivity and one minus the specificity of a “test,” considering the presence of Species A to be a test for the presence of Species B. This is analogous to the use of a medical screening test for the presence of a disease, where this ratio is referred to as the *positive likelihood ratio* (Deeks and Altman 2004). Note also that the reciprocal of OR above is the sensitivity of a test for the absence of Species B scaled by one minus its specificity, so the same statistic is useful for both indication and contraindication.

## Case study – species pairwise association analysis of temperate woodland birds

### Study area

Our case study comprised 134 temperate woodland remnants and 65 replanted woodlands located on 45 farms on the southwestern slopes of New South Wales, southeastern Australia. The predominant form of native vegetation was temperate eucalypt woodland (*sensu* Lindenmayer et al. 2010). Plantings were areas of planted native vegetation characterized by a mix of local endemic and exotic Australian plant species. Most plants in restored areas were typically spaced 2 m apart, but there was not a standard set of spacing and plant species composition protocols applied in revegetation efforts. Our study area spanned the towns of Junee (0552952E 6140128N) in the north, Albury (0494981E 6008873N) in the south (a distance of ∼150 km), and Gundagai (600532E 6119073N) and Howlong (467090E 6017897N) in the east and west, respectively (a distance of ∼120 km) (see Cunningham et al. 2007).

### Bird survey protocols

Our study region supports more than 170 bird species. Over half of these species are woodland dependent and are strongly associated with woodland vegetation cover (Lindenmayer et al. 2012). Our first survey of birds was in 2002, on 164 sites. A further 16 sites were added in 2004 and the remaining 19 in 2006; all 199 sites were then surveyed in 2008, 2009, and 2011. The bird survey procedures (aural and visual observations) were reviewed and approved by the Australian National University's Animal Ethics Committee. Our bird-counting protocols entailed repeated 5-minute point interval counts (*sensu* Pyke and Recher 1983) at each of the 0-m, 100-m, and 200-m points along a permanent transect at each site. In the spring of each of the above years, all sites were surveyed by two different observers on different days. We completed counts between 5.30 and 9.30 am and did not undertake surveys on days of poor weather (rain, high wind, fog, or heavy cloud cover).

We recorded the presence of all birds seen or heard in discrete distance classes at each of the three permanent markers at each site. For this study, we considered a bird to be present at a site if it was recorded by at least one observer on at least one transect point at a radius of not greater than 50 m. We did not attempt to estimate detection rates (MacKenzie et al. 2002), as we had only two observations at each site in each survey, but we note that Welsh et al. (2013) suggest that the current statistical methods for detection and occupancy do not improve model fit, and in some cases, they can make the outcomes worse.

### Statistical methods

Treating each survey as independent, odds ratios can be calculated directly from the observed proportions of individual and paired species. Equivalently, they can be estimated together with standard errors by fitting a logistic regression model for each pair of species, for example, Species A and Species B, and deriving the odds ratio (OR) to assess whether B indicates A from the formula:


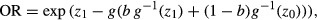


where *b* is the proportion of sites at which B occurred, *g* is the logit transformation, *g*(*b*) = ln(*b*/(1–*b*)), and *z*_0_ and *z*_1_ are the linear predictors from the fitted model for the presence of A in the absence of B and in the presence of B, respectively (we used the RFUNCTION command in the GenStat system; VSNi 2013, which estimates standard errors by the delta method).

In our study, there were repeated measurements at each site, and the resulting correlation can be expected to increase the standard errors. Therefore, we calculated the odds ratios by fitting a generalized linear mixed model for each pair of species, including a random site effect (using the GLMM command in GenStat).

Another complicating issue is the large number of odds ratios considered, which inflates the chance of spurious results. The full set of *n*(*n*–1) ratios for *n* species is strongly intercorrelated and is derived from just *n* variables recording the presence of each species. Therefore, a conservative (Bonferroni) adjustment for multiplicity would compare the *P*-value of each odds ratio against 0.05/*n* to establish the statistical significance of the difference of the odds ratio from 1. A more detailed study of significance could be conducted using approaches such as those in the programs Pairs (Ulrich 2008), Turnover (Ulrich 2012) and Ecosim (Gotelli and Entsminger 2004). However, with the large amount of data from our surveys, individual odds ratios as large or small as our chosen criteria (3 and ⅓) are very likely to be statistically significant even if adjusted for multiplicity. We studied the null distribution of odds ratios (i.e., in the absence of real effects) by simulation, to quantify the likelihood of finding spuriously large associations. Associations with odds ratios less than 3, or greater than ⅓, may also be statistically significant, but we focussed our case study on effects that we considered to be ecologically substantial.

## Results

We illustrate our methodology by assessing bird species associations in woodland remnants. We then compare these with species associations in plantings.

### Woodland remnants

We present an association diagram for the 795 surveys in woodland remnants (Fig. 1). The nodes in the association diagram represent the 38 species that occurred in at least 10% of field surveys at these sites, with each species given a reference number (Table 2). We recorded the presence of another 118 species, ranging in rarity from 50 species recorded five times or fewer in the whole study (<0.3% presence) to some with just less than 10% presence. The arrowed lines indicate the strength and direction of indications (red, representing an odds ratio >3) and contraindications (blue, representing an odds ratio <⅓). For example, the strongest indication was that of the white-plumed honeyeater *Lichenostomus penicillatus* (Ref 34) by the dusky woodswallow *Artamus cyanopterus* (Ref 11). The odds ratio is 13.1, because the white-plumed honeyeater was found at 57% of all sites, compared with 95% of the sites where the dusky woodswallow was found. In contrast, there was “perfect” contraindication (black line) between the grey butcher-bird *Cracticus torquatus* (Ref 14) and restless flycatcher *Myiagra inquieta* (Ref 25), because these two species never co-occurred.

**Table 2:** Species present in at least 10% of surveys and % presence in remnants and plantings

			% Presence	
			
Ref	Species common name	Species scientific name	Remnants	Plantings
1	Australian magpie	*Cracticus tibicen*	84	74
2	Australian raven	*Corvus coronoides*	16	14
3	Black-faced cuckoo-shrike	*Coracina novaehollandiae*	30	24
4	Brown treecreeper	*Climacteris picumnus*	29	3
5	Cockatiel	*Nymphicus hollandicus*	12	3
6	Common bronzewing	*Phaps chalcoptera*	12	9
7	Common starling	*Sturnus vulgaris*	48	37
8	Crested pigeon	*Ocyphaps lophotes*	43	44
9	Crested shrike-tit	*Falcunculus frontatus*	13	12
10	Crimson rosella	*Platycercus elegans*	12	11
11	Dusky woodswallow	*Artamus cyanopterus*	18	4
12	Eastern rosella	*Platycercus eximius*	79	59
13	Galah	*Eolophus roseicapillus*	59	33
14	Grey butcher-bird	*Cracticus torquatus*	12	5
15	Grey shrikethrush	*Colluricincla harmonica*	34	43
16	Jacky winter	*Microeca fascinans*	12	2
17	Laughing kookaburra	*Dacelo novaeguineae*	21	6
18	Little friarbird	*Philemon citreogularis*	15	8
19	Magpie-lark	*Grallina cyanoleuca*	45	33
20	Noisy miner	*Manorina melanocephala*	66	27
21	Peaceful dove	*Geopelia striata*	12	8
22	Pied butcher-bird	*Cracticus nigrogularis*	16	3
23	Red wattlebird	*Anthochaera carunculata*	19	44
24	Red-rumped parrot	*Psephotus haematonotus*	58	54
25	Restless flycatcher	*Myiagra inquieta*	11	3
26	Rufous songlark	*Cincloramphus mathewsi*	41	49
27	Rufous whistler	*Pachycephala rufiventris*	14	35
28	Sacred kingfisher	*Todiramphus sanctus*	11	3
29	Striated pardalote	*Pardalotus striatus*	68	48
30	Superb fairy-wren	*Malurus cyaneus*	13	61
31	Superb parrot	*Polytelis swainsonii*	14	8
32	Welcome swallow	*Hirundo neoxena*	12	13
33	White-browed woodswallow	*Artamus superciliosus*	18	15
34	White-plumed honeyeater	*Lichenostomus penicillatus*	57	75
35	White-winged chough	*Corcorax melanorhamphos*	29	20
36	White-winged triller	*Lalage sueurii*	16	12
37	Willie wagtail	*Rhipidura leucophrys*	61	79
38	Yellow-rumped thornbill	*Acanthiza chrysorrhoa*	12	34

**Figure 2:**
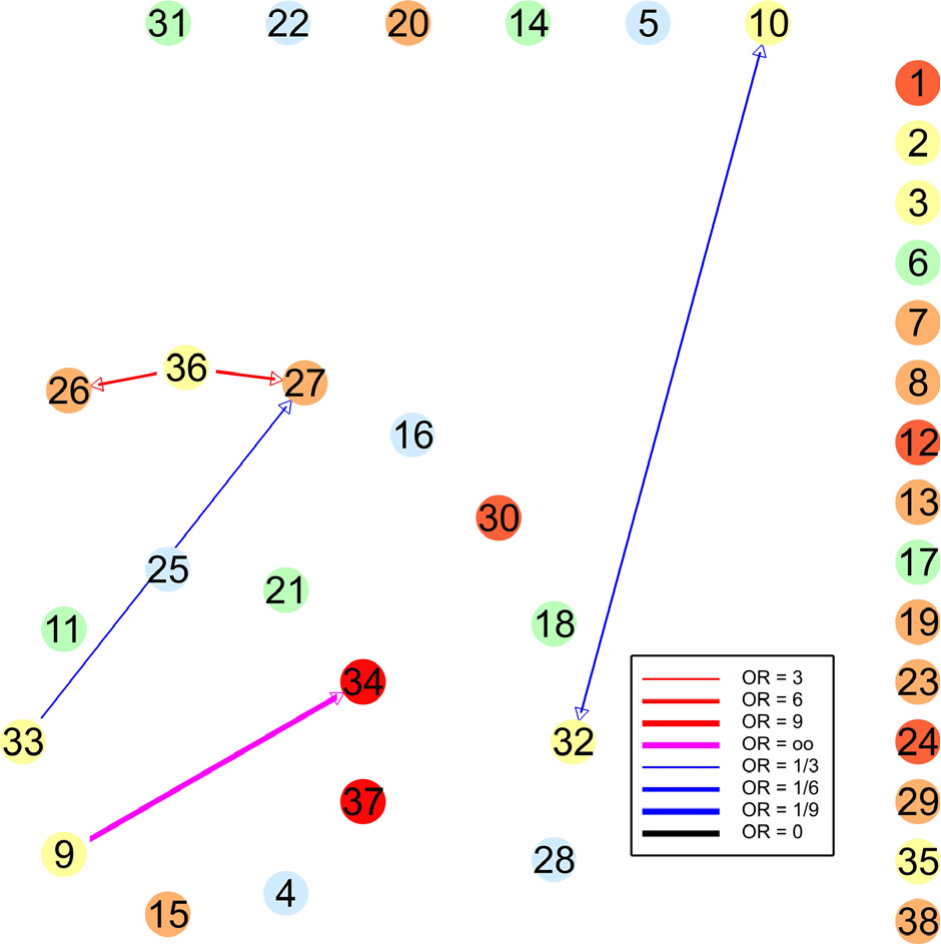
Association diagram for plantings (345 surveys); key as for Fig. 1.

The arrangement of the nodes in Fig. 1 shows a cluster of nine species, all of which are positively associated with at least half the other species in the cluster. The white-plumed honeyeater (Ref 34) and willie wagtail *Rhipidura leucophrys* (Ref 37) were indicated by many species, but did not indicate other species because they were common. Several other species were positively associated with one or two of these nine species, or in pairs or chains, but there are no other clear clusters. To facilitate the comparison with Fig. 2, we arranged these species around the cluster together with other species that are positively associated with the cluster in that figure. There were 15 species with no associations >3 or <⅓. All the odds ratios represented by red lines in Fig. 1 were individually significantly different from 1 (largest *P*-value = 0.008), as were all but one of the odds ratios represented by blue lines (*P* < 0.05). The exception was the contraindication of the peaceful dove *Geopelia striata* by the superb parrot *Polytelis swainsonii* (Refs 21 and 31; *P* = 0.08). Table 3 lists all the odds ratios. We studied the distribution of odds ratios by simulation, in the absence of real effects (for details, see Appendix 2), and typically found only two spuriously large odds ratios and no spuriously small ones that were individually statistically significant (of 1406 odds ratios).

**Table 3:** Odds ratios illustrated in Fig. 1, with 95% confidence interval and unadjusted approximate *P*-values for test of difference from 1, for association of species at remnant sites; Ref 1 refers to the species that is indicated or contraindicated by the species with Ref 2

			95% CI		
				
Ref 1	Ref 2	OR	Lower	Upper	*P*-value
34	11	13.15	3.81	45.31	<0.001
34	4	10.43	4.91	22.14	<0.001
34	21	9.60	2.68	34.33	<0.001
20	14	7.44	1.70	32.64	0.008
37	9	6.29	2.36	16.77	<0.001
34	9	6.20	2.07	18.59	0.001
34	16	5.77	2.04	16.36	0.001
37	11	5.70	2.42	13.44	<0.001
37	28	5.36	2.01	14.26	<0.001
37	25	5.11	1.83	14.30	0.002
37	4	5.09	2.84	9.13	<0.001
36	27	4.52	3.23	6.31	<0.001
15	25	4.51	2.23	9.14	<0.001
34	25	4.41	1.55	12.51	0.005
15	21	4.35	2.30	8.24	<0.001
15	4	4.35	3.14	6.03	<0.001
37	33	4.26	2.15	8.45	<0.001
37	30	4.17	1.93	9.00	<0.001
37	21	4.14	1.75	9.79	0.001
34	15	4.10	2.55	6.60	<0.001
34	33	4.00	2.02	7.92	<0.001
4	16	3.95	2.07	7.56	<0.001
37	15	3.89	2.47	6.13	<0.001
27	36	3.86	2.79	5.34	<0.001
33	9	3.72	2.59	5.33	<0.001
20	22	3.70	1.53	8.92	0.004
4	11	3.63	2.12	6.23	<0.001
34	18	3.60	1.83	7.12	<0.001
37	18	3.50	1.75	7.00	<0.001
11	4	3.49	2.87	4.25	<0.001
9	4	3.48	2.98	4.07	<0.001
34	30	3.43	1.62	7.27	0.001
4	25	3.41	1.67	6.95	<0.001
36	25	3.37	2.24	5.06	<0.001
4	9	3.35	1.83	6.13	<0.001
33	11	3.32	2.45	4.48	<0.001
34	32	3.26	1.53	6.95	0.002
9	11	3.19	2.36	4.31	<0.001
16	4	3.16	2.64	3.78	<0.001
9	33	3.15	2.35	4.22	<0.001
21	4	3.01	2.52	3.59	<0.001
34	22	0.33	0.20	0.56	<0.001
26	31	0.33	0.19	0.58	<0.001
14	33	0.33	0.12	0.94	0.039
36	14	0.33	0.13	0.81	0.016
22	4	0.31	0.17	0.57	<0.001
9	22	0.30	0.11	0.84	0.022
28	22	0.29	0.09	0.94	0.040
22	9	0.28	0.10	0.77	0.014
18	5	0.27	0.10	0.74	0.011
31	21	0.25	0.05	1.16	0.077
30	22	0.25	0.08	0.82	0.022
33	14	0.25	0.09	0.70	0.009
5	18	0.24	0.09	0.64	0.005
22	28	0.24	0.07	0.78	0.018
25	22	0.24	0.06	0.97	0.045
21	31	0.21	0.05	0.93	0.041
31	16	0.20	0.04	0.94	0.041
22	30	0.20	0.06	0.64	0.007
16	31	0.18	0.04	0.80	0.024
22	25	0.18	0.04	0.75	0.018
14	18	0.11	0.01	0.78	0.028
11	22	0.11	0.02	0.47	0.003
22	11	0.09	0.02	0.37	<0.001
18	14	0.08	0.01	0.63	0.016
14	25	0.00	0.00	*	*
25	14	0.00	0.00	*	*

* Upper limit and *P*-value are not available for estimates equal to 0.

### Plantings versus woodland remnants

The pattern of species presence and association in planted sites contrasted markedly with that in the woodland remnants (Fig. 2). Figure 2 displays this in an association diagram, using the same layout of nodes as adopted for the remnants, to facilitate comparison (the odds ratios are listed in Table 4). Many species were far less prevalent in plantings than remnants: There were 13 species with <10% presence (coded blue or green), and we therefore excluded assessment of any associations with them. Conversely, we note that 10 species were more common in plantings than in remnants, such as the superb fairy-wren *Malurus cyaneus* (Ref 30), which was present 61% of the time in plantings compared with 13% in remnants.

**Table 4:** Odds ratios illustrated in Fig. 2 and unadjusted approximate *P*-values for test of difference from 1, for association of species at planting sites; Ref 1 refers to the species that is indicated or contraindicated by the species with Ref 2

			95% CI		
				
Ref 1	Ref 2	OR	Lower	Upper	*P*-value
34	9	∞	0.00	*	*
27	36	4.23	1.83	9.79	<0.001
26	36	4.14	1.92	8.93	<0.001
10	32	0.33	0.08	1.39	0.131
32	10	0.32	0.07	1.39	0.128
27	33	0.32	0.15	0.69	0.004

* Upper limit and *P*-value are not available for estimates equal to 0.

In contrast to the remnants, the plantings were characterized by no clusters of species and far fewer associations. Eight of the indications shown in Fig. 1 between species not rare in either habitat are not apparent in Fig. 2, compared with only one new indication in Fig. 2. Six of the missing indications were of the white-plumed honeyeater or willie wagtail (Refs 34 and 37), both of which were more common in plantings (75 and 79% on plantings, respectively, compared with 57% and 61% on remnants).

## Discussion

A major goal of ecology is to identify and understand the patterns and drivers of species associations. This includes the need to identify mechanisms underpinning patterns in ecological networks to better understand community dynamics (Proulx et al. 2005; Bascompte 2010). We have demonstrated how our approach for exploring and quantifying species pairwise associations enables this to be done *in silico* in the context of species assemblages. Understanding associations between species can help better quantify interlinked extinctions (Saterberg et al. 2013) and potential losses that might occur if particular species are removed leading to the losses of codependent or closely associated species (sometimes termed coextinction cascades; Koh et al. 2004; Bascompte 2009). Better understanding is also critical for quantifying the effectiveness of restoration activities (as shown in our case study; see Fig. 2). Determining the strength of associations is also important because it can indicate which species may be those most vulnerable to decline or extinction if a network is disrupted (Saavedra et al. 2011) and conversely how network architecture can influence other processes such as competition (Bastolla et al. 2009). Finally, our approach has significant potential application in conservation because ecologists need to focus not only on maintaining species, but also on conserving species interactions (Tylianakis et al. 2010).

Our new approach for examining species pairwise associations goes beyond simple descriptions of the count, identity, or abundance of species, as does the approach of Ovaskainen et al. (2010). Both allow the exploration of patterns of association and the way the patterns change with key factors such as vegetation type (as in our example), or habitat structure, season, and the co-occurrence of dominant species (either positive or negative). These approaches therefore enable informative comparisons between species assemblages in different environments. Our approach also enables exploration not only of direct association effects between pairs of species, but also of the impacts of second-order associations, which become apparent when a dominant species is removed, such as a reverse keystone species (*sensu* Montague-Drake et al. 2011). This can be achieved by comparing the odds ratios from two different analyses of species pairwise associations, one for sites where the dominant species occurs and one for sites where it does not. Notably, many previous studies quantifying the strength of associations between species have typically been within individuals of the same species (Mersch et al. 2013) or a small number of species (Estes et al. 2011), rather than the bulk of a species-rich assemblage (but see Tylianakis et al. 2007; Gotelli and Ulrich 2010; Steele et al. 2011; Veech 2013). Our approach is capable of being applied to large numbers of pairwise associations without massive computational resources and therefore allows the examination of highly diverse assemblages.

### Examining characteristics of networks of species pairwise associations

The results of our case study appear to share many of the features of other kinds of networks such as plant–animal mutualistic networks (reviewed by Proulx et al. 2005; Bascompte 2009). One such feature is “heterogeneity,” in which the bulk of the species interact with a few species and a few species have a much higher number of interactions than would be expected from chance alone. This is depicted in Fig. 1, which shows that two species (the white-plumed honeyeater and willie wagtail) were positively associated with many other species, and one (the pied butcher-bird) was negatively associated with many. In contrast, nearly half of the species do not have strong associations with any others. We also found evidence in Fig. 1 of “compartmentalism” (Bascompte 2010), with nine species more strongly associated with each other than with other species in the assemblage. Another feature of networks of species is the occurrence of “asymmetric links.” We also found evidence of these; for example, the dusky woodswallow was strongly associated with the white-plumed honeyeater in the sense that the second species nearly always occurred when the first did (Fig. 1). However, the reverse was not the case.

### Explanation of the key findings in our case study

There are many underlying reasons for associations between species. Functionally similar or closely related taxa might be adapted to similar environments or gain mutual benefits; for example, enhanced foraging opportunities can result in mixed-species feeding flocks and produce a greater number of species associations (Bell 1980; Sridhar et al. 2012). Species may also share similar nesting requirements or predator avoidance strategies, thus resulting in positive associations. Species might also choose habitat using information gleaned from other species present at a location (Smith and Hellman 2002), particularly a species that is very similar to itself (Seppanen et al. 2007). However, functional similarity might also result in negative associations due to competition (e.g., see Lovette and Hochacka 2006) or interference (Mac Nally et al. 2012). Our new approach can be used to identify the direction of associations between species and to help generate hypotheses for further testing about community assembly and structure.

The differences we found in the pattern of species association between remnants and plantings (Fig. 1 vs. Fig. 2) mostly involved the white-plumed honeyeater or willie wagtail, both of which were more common in plantings. The absence of indications of these species by others (except the crested shrike-tit *Falcunculus frontatus*) may be a result of their being more common, and contraindicating species less common, in remnant sites. There are major differences in the structure and plant species composition of these two kinds of vegetation (Lindenmayer et al. 2012), as reflected in large differences in stem density between plantings and woodland remnants.

## Conclusions

We present a new method of analysis which can provide insights into patterns of species association that goes well beyond simple ordination and other kinds of traditional compositional analyses about the identities of taxa in a given assemblage occurring across a number of sites. Our approach enables associations between many species to be explored simultaneously in a network association diagram, while remaining computationally feasible. This helps generate a new understanding of the influence of factors that affect the sign, direction, and magnitude of species associations, such as vegetation type, habitat attributes, and season. The method also allows the exploration of cascading second-order associations in the presence or absence of a key individual species. This opens up a range of new possibilities to explore the processes that determine the structure and composition of ecological communities.

## Appendix 1: Rules to guide construction of a network diagram

1. Identify the largest group of three or more species in which all pairs are positively associated; that is, for a pair of species A and B in the group, either A indicates B, or B indicates A, or both.
2. Find that species, of those that remain, with the largest number of positive associations with the existing group, and add it to the group if it is associated with 50% or more of the species in it.
3. Repeat (2) until no further species can be found to add to the group, dropping out at each stage any species that is no longer associated with 50% of the others in the group.
4. Arrange the resulting cluster of species as a regular polygon in the bottom left corner of the graphical frame, with species represented by colored circles at the vertices, color-coded according to commonness of the species.
5. Repeat (1–3) until no further clusters of three or more species can be found; if a new cluster consists of just one species plus several in an existing group, define the cluster as the one species plus those in the existing group to which it is associated; if two identified clusters share some species, display the smaller group as an irregular polygon attached to the larger, using the same distance between vertices as far as possible.
6. For each remaining species with a positive association with some species in a cluster, represent it as a point outside the cluster, but further away from each point in the cluster than the distance between points in the cluster.
7. For each species with a negative association with some species in the largest cluster, represent it as a point in the top half of the frame.
8. Draw red lines between all pairs of species where one indicates the other, with an arrow to indicate direction and with line thickness proportional to the odds ratio.
9. Draw blue lines similarly to represent contraindication, with thickness proportional to the reciprocal of the odds ratio.
10. Arrange assignment of species to points in a cluster, and position of outlying points, to reduce crossover of lines.
11. To avoid very thick lines, use moderately thick black lines for contraindications where species do not co-occur and moderately thick magenta lines where species always co-occur.

## Appendix 2: Simulation study of the effect of multiplicity on significance of odds ratios

We investigated the chance of spurious effects for the analysis of surveys on Fig. 1. Using the observed proportions of sites at which each of the 38 nonrare species was present, we generated random patterns of presence at each site–survey combination in our dataset, for each species independently, that is, under the null hypothesis of no indication or contraindication effects. We included a random site effect with variance set equal to the mean of the variances (1.16) from the GLMMs fitted to the observed data. For each of 1000 simulations, we fitted GLMMs to the random data as for the observed data and counted the number of odds ratios greater than 3 (indications) or less than ⅓ (contraindications). The median number of indications was 2, and the 97.5 percentile was 7, while there were no contraindications at all in any of the simulations. This asymmetry is likely to be a consequence of the fairly large random effect. Repeating the exercise, but allowing the variance of the random effect also to vary from simulation to simulation (modeled on the distribution of variances estimated for each pair of species for Fig. 1), made little difference to the number of large or small odds ratios (the 97.5 percentile for the number of indications was 8 rather than 7). A final set of simulations with no random site effect gave the median number of both indications and contraindications as 0, with 97.5 percentile 1 and 4, respectively. Relaxing the criterion for indications to 2.5, and for contraindications to 0.4, resulted in a median of 13 indications, with 97.5 percentile 40 (still with no contraindications). So this lower criterion gives rise to an unacceptably high number of spurious indications for our dataset.
